# Exposing Face Manipulation Based on Generative Adversarial Network–Transformer and Fake Frequency Noise Traces

**DOI:** 10.3390/s25051435

**Published:** 2025-02-26

**Authors:** Qiaoyue Man, Young-Im Cho

**Affiliations:** Department of Computer Engineering, Gachon University, 1342 Seongnamdaero, Sujeong-gu, Seongnam-si 13120, Republic of Korea; manqiaoyue@gmail.com

**Keywords:** generative adversarial network, face manipulation techniques, deepfake generation, forgery detection

## Abstract

In recent years, with the application of GANs and diffusion generative network algorithms, many highly realistic synthetic images are emerging, greatly increasing the potential for misuse, and deepfakes have become a serious social concern. To cope with indistinguishable deep forgery face images, this paper proposes a novel detection network with a generative adversarial network (GAN) and transformer as the main architectures. It adds frequency domain analysis and noise detection prediction modules. In the proposed model in which GAN is used to capture local forgery, artifacts and transformers are used to model global dependencies and predict anomalies in the forged images using frequency domain and noise information; the framework enhances the detection of subtle and diverse deep forgery patterns. Experiments on benchmark datasets show that the proposed method achieves higher accuracy and robustness compared to existing methods.

## 1. Introduction

As image generation networks based on generative adversarial networks (GANs) [1] and diffusion networks emerge at an astonishing rate, hundreds of millions of forged synthetic images have appeared on the Internet and in our daily lives, bringing surprises and convenience to people, but also causing indistinguishable confusion and security risks. In particular, with the emergence of deepfake technology, such forged face images can replace the target face with the source face or continue to camouflage the source face with feature modifications to generate fake face images indistinguishable from the human eye. Using this technique, an attacker can easily forge others or create high-quality images and video information of non-existent people for illegal, political, or commercial purposes, posing a serious threat to social security and personal privacy. Unlawful people may use the forged information for a variety of malicious purposes, including dissemination of false information, invasion of privacy, creation of social polarization, and creation of conflicts. To mitigate the misuse of deep forgery face technology, the development of appropriate detection algorithms is imminent.

The combination of deep learning with computer vision techniques and the exponential increase in computer arithmetic power has opened the door to producing hyper-realistic fake images and videos. Currently, generative network-based models are very powerful in producing deepfakes, which are difficult to distinguish by traditional methods. For example, face-swapping software such as DeepfaceLab [2], Fsceswap [3], Facefusion [4], and face-changing technology based on stable diffusion model [5,6], which are based on deep learning, including convolutional neural networks and self-encoders, are used to train and generate highly realistic face-swapping models. It is possible to replace one person’s face with another’s and generate images and videos with such a highly realistic effect that even humans have trouble distinguishing what is fake and what is real. As a result, more and more researchers have begun to realize the necessity of deepfake detection research, and in recent years, the research on the generation and detection of deepfakes has been growing rapidly in the computer vision and machine learning communities.

In the early stages of deepfake detection research, generally, forged faces expose some visually obvious clues such as high variability in facial texture, inconsistent color distribution, artifacts, etc. The traditional approach [7] of using the corresponding handcrafted features extracted from the pixel statistics of intrinsic face images and training shallow classifiers is very effective for the detection of these forged images. This is due to the fact that the handcrafted features are more targeted, and the detector can be trained in a targeted manner based on the specific features exposed by the image. With the iteration of deepfake technology, based on the continuous evolution of generative adversarial networks, the generated faces have become so realistic that they are difficult to detect with the naked eye, making it difficult to distinguish the authenticity of the images. Therefore, it is crucial to use the powerful feature expression capabilities of deep convolutional learning to learn the subtle discriminative feature information implicit in the forged samples, which is impossible with traditional methods. Common approaches based on deep convolutional methods [8,9,10] are extracting video frames and performing feature extraction computation on the images using multilayer convolutional operations with fully connected layers, followed by identifying and classifying true and false images. However, these simple methods with just convolutional feature computation rely on surface feature correlation to distinguish real images by focusing on the information within each receptive field. These methods expose the drawbacks of performance degradation when using face data other than that of the training dataset for detection. To mitigate this drawback, some studies have incorporated an attention mechanism model [11] into CNN-based deep forgery detection algorithms [12,13] to improve performance within and across datasets by enlarging the region of local image features. However, this method is powerless in the face of high-quality fake face image data generated through a large amount of training. For this reason, a large number of researchers have recently used GAN to regenerate fake images to explore the existence of fake traces [14], especially the natural sensitivity to image lighting, reflection, texture, and edge blur that other models lack, greatly enhancing the detection ability. Although these methods [15,16] perform well in detecting large-resolution fake images, their model generalization ability is weak and is only suitable for the detection task of fake face images generated using GAN.

As deepfake technology becomes more efficient and sophisticated and produces more and more realistic images, many existing deepfake detection models have become too weak to accurately differentiate between real and fake faces in their images. Faced with this challenging task, in this paper, we propose a novel detection framework with generative adversarial networks (GANs) and transformers [17] as the main architectures. This adds frequency domain [18] analysis and noise detection prediction modules. In the proposed model in which GANs are used to capture local forgery artifacts and transformers are used to model global dependencies and predict anomalies in the forged images using frequency domain and noise information, the framework enhances the detection of subtle and diverse deep forgery patterns. The specific contributions are as follows:

1. We introduce a novel fusion framework for deepfake face feature detection—FFC. The framework mainly consists of three modules: GAN and transformer image feature extraction module, frequency domain and noise feature extraction module, and feature true and false identification classification module. The detected face image’s global and local feature coherence and consistency are used to enhance the forgery detection ability.
2. We use the reconstructed GAN and transformer blocks to extract features from fake face images. ResNet with added dilated convolutions is used as a generator to generate fake images. Global and local convolutional networks are used as discriminators, and transformer blocks are connected to identify features of the generated fake images, improving the ability to identify subtle features of fake images.
3. We designed a frequency domain and noise feature detection module to detect frequency domain anomalies and noise discrepancies present in the forged images.
4. Our designed FFC fusion network model for deep forgery face image detection performs more efficiently and robustly than other good models in tests based on FF++, Celeb-DF, and DFDC datasets.

## 2. Related Works

### 2.1. Deepfake Methods

Benefiting from the advancement of deep learning, deepfake technology [19] has been rapidly developed. Justus et al. [20] proposed the face2face network, a method for real-time face reconstruction from monocular target video sequences, which transfers facial expressions from a source face to a target face by fitting a 3D deformable face model to two faces. Li et al. [21] proposed the FaceShifter algorithm, a two-stage framework to achieve high-fidelity and occlusion-aware face-changing technology. It extracts target attributes at various spatial resolutions through a multi-level attribute encoder, and the generator adaptively integrates the face identity and attributes when synthesizing the face to generate a highly realistic replacement face. Yuval et al. [22] proposed Face Swapping GAN (FSGAN) for face swapping and reproduction; the network is based on Recurrent Neural Network (RNN), combining Poisson optimization with Poisson mixing loss, and using a face fusion network to achieve the seamless fusion of two faces while preserving the target skin color and lighting conditions, which is an excellent performance in both image and video face forgery tasks. Chen et al. [23] proposed a SimSwap network with an ID Injection Module (IIM). This module transfers the identity information of the source face into the target face at the feature level and implicitly preserves the target face’s facial attributes, greatly enhancing the forgery capability.

### 2.2. Face Forgery Detection

Previous research on deep forgery face image detection has focused on using traditional statistical analysis of image features, and feature rule detection, such as Fourier transform-based image frequency domain feature analysis, edge analysis, color and artifact analysis, and other methods. These methods usually do not rely on deep learning models but are based on the intrinsic properties of the image, which are analyzed by feature engineering and algorithms to determine image authenticity. Kim et al. [24] proposed to compute the diffusion velocity using a total variation (TV) flow scheme and extract anti-spoofing features based on the local patterns of the diffusion velocity, known as localized velocity patterns (LSPs), to train linear SVM classifiers for the detection of true and false images. Li et al. [25] used the difference in the statistical features of true and false images in terms of the chromaticity component of HSV and YCbCr color space to distinguish between deepfake images. Lugstein et al. [26] developed a PRNU-based facial deformation and facial modification detection method by applying the popular PRNU (Photo Response Non-Uniformity) detection method in image forensics to deep forgery detection. These traditional methods have difficulties in adapting to the detection of high-quality forged images and lack robustness to specific forgery techniques. Recently, many methods using deep neural networks to capture modified features hidden in forged images have shown impressive impressions. Yu et al. [27] proposed a Patch-DFD depth forgery detector, a block-based Facial Block Mapping (FPM) solution to acquire multiple part-based feature maps to maximize the original details of each facial block, and the addition of a BM-pooling module to reduce quantization error and improve recognition accuracy. Alkishri et al. [28] analyzed the high-frequency Fourier patterns of real and deep network-generated images. They used the relative size and attenuation rate of the high-frequency spectrum to reproduce the high-frequency features with observable systematic defects, thereby distinguishing forged images. Zhao et al. [29] designed a new framework TAN-GFD based on texture information and adaptive noise mining, which combined the pixel intensity and gradient information on the feature map to extract multi-scale texture difference features from different shallow feature maps, thereby improving the generalization ability of the model detection. Kohli et al. [30] proposed a method to detect forged facial images by using a two-dimensional global discrete cosine transform (2D-GDCT) to convert it into the frequency domain and using a three-layer frequency convolutional neural network (fCNN), which further improved the detection performance of forged facial images. Through our research, we found that most of the previous methods used a single CNN model or image frequency detection method, as well as an improved GAN model method. Although these methods have greatly improved the performance of deepfake image detection, they are still unable to cope with the increasingly realistic fake face images at this stage. For example, the CNN-based method cannot accurately identify fake face images generated by GAN and stable diffusion models, and the GAN-based method only performs well in detecting fake images generated by specific models. In the face of these problems, we proposed a new fusion framework—FFC. The GAN and transformer combined with the image feature extraction module solve the problem that GAN is single and specific and can only detect specific generated fake images. The addition of the image frequency domain and noise feature extraction module captures the residual traces of fake generated by the image, further enhancing the model’s forgery detection ability.

## 3. Materials and Methods

**Figure 1:**
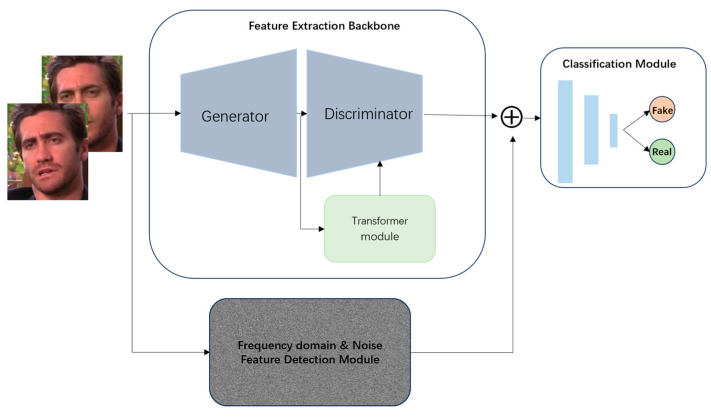
FFC network framework.

Our proposed FFC network framework fusions three main modules, as shown in Figure 1. The framework contains GAN combined with a transformer block for the feature extraction backbone, a frequency domain and noise feature detection module, and a forged image classification module.

### 3.1. Feature Extraction Backbone

**Figure 2:**
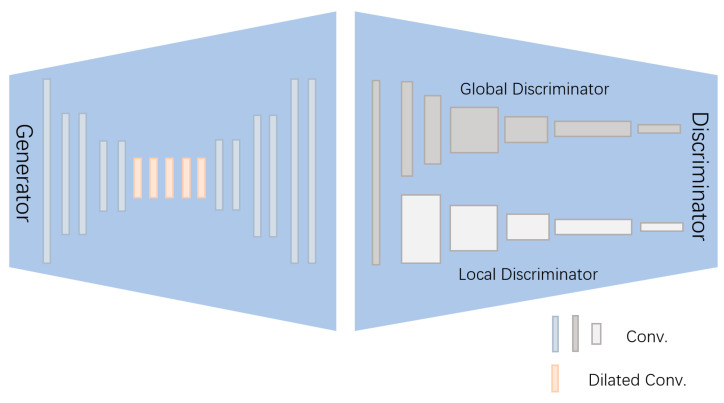
GAN-based forgery feature detection module.

In the feature extraction backbone, we improve the internal framework based on GAN and add transformer blocks, as shown in Figure 2, to capture the complex spatial, texture, and contextual features in the fake images.

The *G* (generator) uses ResNet with added dilated convolutions. The convolution operation of ResNet can extract low-level features of edges and textures. By adding dilated convolutions based on ResNet, the generator can capture a larger receptive field and capture local and global contextual information. The generator can generate detailed intermediate features at each layer, and the generated forged images *x_fake_* further amplify the hidden forgery traces of the original image. The *D* (discriminator) consists of a global neural network and a local neural network, which capture the global *f_global_* and local features *f_local_* of the forged image, respectively, through the combination of the two features, to detect, in more detail, the overall lighting consistency and the forged anomalies in the face-specific eye, nose, and mouth regions in the forged image. Global and local features are obtained by weighted fusion:

(1) $$F_{\mathrm{dis}\;}=\alpha F_{\mathrm{global}\;}+\beta F_{\mathrm{local}\;}$$

As with the standard GAN, the adversarial loss between the generator and the discriminator is:

(2) $$L_{G}=-E_{z∼p_{z}(z)}[log(D(G(z)))]\;L_{D}=-E_{x_{\mathrm{real}\;}∼p_{\mathrm{data}\;}}\left[\log\left(D\left(x_{\mathrm{real}\;}\right)\right)\right]-E_{z∼p_{z}(z)}[log(1-D(G(z)))]$$

**Figure 3:**
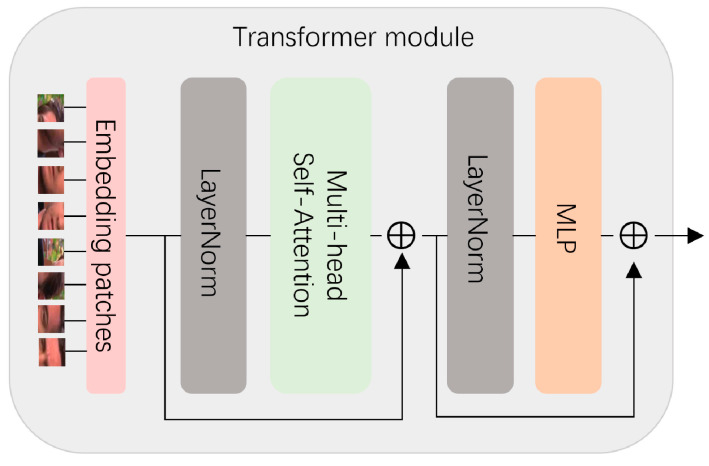
Transformer module.

Due to the pattern specificity of GAN, it lacks a certain generalization ability for forged image detection. Here, we add a transformer module, and use the original framework’s transformer module, as shown in Figure 3. This module includes an MLP layer, a multi-head self-attention layer, a layer-norm layer, etc., which divides the forged face image into 8 patches. Each patch can be regarded as a language “word” similar to NLP (natural language processing). The multi-head attention feature extracts the information in each patch, obtains the global feature information through multi-layer calculation, and determines the forged feature part. Use the self-attention mechanism to improve the global perception of forged features. Improving detection of non-GAN forged images via fusion. The transformer block focuses on capturing global long-range dependencies. It is connected in parallel with the discriminator to provide additional global features *f*_trans_. Combining the global neural network, local neural network, and transformer blocks, the discriminator’s feature fusion is as follows:

(3) $$F_{\mathrm{combined}\;}=W_{\mathrm{fusion}\;}\left[F_{\mathrm{dis}\;},F_{\mathrm{trans}\;}\right]$$

### 3.2. Frequency Domain and Noise Feature Prediction Module and Classification Module

**Figure 4:**
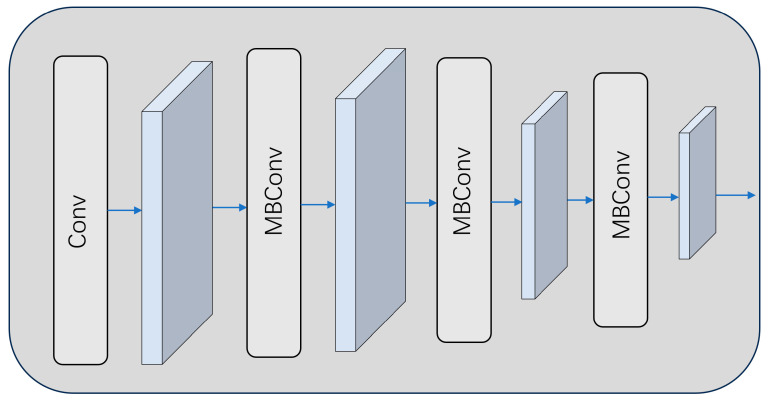
Frequency domain and noise feature detection module.

During the generation process deepfake face images, abnormal image frequency domain, and noise characteristics will be produced. These characteristics are significantly different from the statistical characteristics of real images, and a single model cannot accurately and effectively detect them. As shown in Figure 4, to capture these hard-to-detect traces of forgery, we added EfficientNet-based frequency domain and noise feature detection modules. First, the input forged image is preprocessed. Fast Fourier transform converts the input image from the spatial domain to the frequency domain to obtain the frequency domain feature map. At the same time, Gaussian filtering extracts the noise residual of the image to obtain the noise feature map. The obtained data are input into the frequency domain and noise feature detection module based on EfficientNet for joint training.

Among them, the fast Fourier transform image feature extraction formula is

(4) $$F\left(u,v\right)=FFT\left(f\left(x,y\right)\right),\;\;R_{hl}=\frac{\sum_{(u,v)\in H}|F(u,v){|}^{2}}{\sum_{(u,v)\in L}|F(u,v){|}^{2}}$$

Gaussian filtering extracts noise, and the formula is

(5) $$\mu_{N}=\frac{1}{M\times N}\sum_{x,y}N(x,y),\sigma_{N}^{2}=\frac{1}{M\times N}\sum_{x,y}{\left(N(x,y)-\mu_{N}\right)}^{2}$$

Finally, the frequency domain features extracted by FFT and the image noise features are fused to form a joint feature vector:

(6) $$F_{FN}=\left[R_{hl},\mu_{N},\sigma_{N},\ldots \right]$$

The classification module includes a feature fusion layer and an SVM classifier. The features extracted by the feature extraction module of GAN combined with the transformer block are fused with the features extracted by the frequency domain and noise feature detection module to obtain a unified feature vector *Z(x)*. The fused features are input into the support vector machine (SVM) classifier to classify the features as true or false and output the final result.

(7) $$Z(x)=[F_{\mathrm{combined}\;},\;F_{FN}]\in R^{1+n}$$

Among them, $Z\in R^{1+n}$ is a two-dimensional vector, representing the feature representation of image x.

## 4. Results

### 4.1. Datasets and Implementation Details

In the experimental validation, three benchmark datasets commonly used in deep forgery face detection tasks at this stage, namely, Celeb-DF, FaceForensi c++, and DFDC, are selected to evaluate the efficiency and robustness of our proposed framework. As shown in Table 1, Celeb-DF [31] is a large-scale challenging dataset for deepfake forensics. The dataset includes 590 original videos collected from YouTube and 5639 fake videos generated by deepfake, with people of different ages, races, and genders in the videos. FaceForensic++ (FF++) [32] is a forensics dataset consisting of 1000 original video sequences manipulated with four automated face manipulation methods: deepfakes, Face2Face, FaceSwap, and NeuralTextures. The data have been sourced from 977 YouTube videos and all videos contain a trackable mostly frontal face without occlusions, enabling automated tampering methods to generate realistic forgeries. DFDC [33], the deepfake detection challenge dataset is a large dataset released by Meta to measure the progress of deepfake detection technology. This dataset is a deep face detection dataset consisting of more than 100,000 fake videos created from 19,154 real videos and fully considers the diversity of subjects and backgrounds in real scenes (skin color, gender, lighting conditions, etc.).

For deepfake detection, the video dataset needs to be preprocessed. Here, we refer to the related experiments of FaceShifter and use RetinaFace [34] to align and crop faces. The cropped image size is 256 × 256, covering the entire face and some background areas. Each video is uniformly sampled and processed at a rate of 10 frames/s. The aligned faces are also manually checked to prevent detection errors. After data cleaning, all corresponding frame images in the video are processed and extracted for testing. In the model setting, in the feature extraction backbone, the learning rate of training is uniformly set to 0.00001 and trained for 1000 rounds. The initial learning rate of the frequency domain and noise detection module based on EfficientNet is 0.256, which is decayed to 0.97 times every 2.4 rounds of iteration and trained for 100 rounds. All experiments are trained on a server equipped with an NVIDIA RTX3090 GPU, and AMD Threadripper 2950x CPU and 64G RAM.

### 4.2. Evaluation Metrics

**Figure 5:**
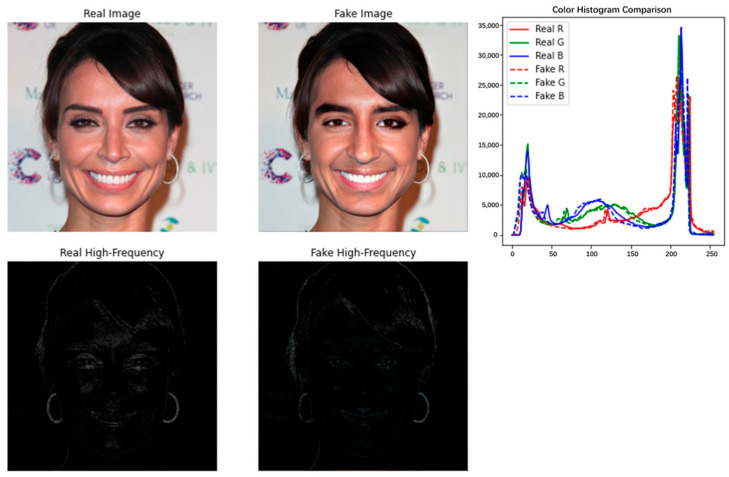
Comparison of noise feature maps and color histograms of real and fake images.

At this stage, the depth of the forged face image has solved the problems of abnormal generation of facial features, blurred face after forged modification, insufficient feature fusion, tearing, light tearing, picture granularity, etc., which existed in the previous forged image. Although the problem of deception of human vision has been solved, some hidden traces of forged operations can still be found under high-performance computer computation. As shown in Figure 5, the curves of the fake image and the real image almost completely overlap in the comparison of the image color histograms, but the blurring of the operative part of the fake face can still be easily detected in the computed image high noise comparison graph.

**Figure 6:**
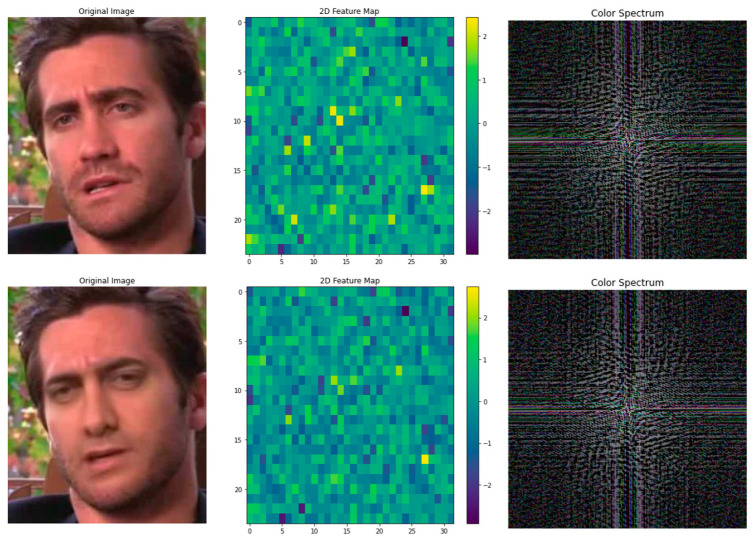
Comparison of 2D features and frequency domain information about real and fake face images.

Based on the celeb-DF dataset, we extract the corresponding face images from the video and use the frequency domain features and 2D feature comparison experiments. We can still find some feature differences, as shown in Figure 6.

In the celeb-DF video dataset-based test, we randomly selected 500 real and fake videos from the dataset in order to conduct the experiment more fairly. Due to the different duration of each video, there may be some performance differences; here, we use the test mean value to count the final performance, and we use the accuracy value and the area under the characteristic curve (AUC) value as the comparison index. As shown in Table 2, our model shows more excellent performance and robustness compared to other video detection models in conducting real-time tests.

We extracted frames from three different types of fake video datasets and compared their comprehensive performance with other most representative excellent models. Compared with the traditional GAN model, although the network model we proposed contains multiple modules and the model size is relatively increased, due to the use of a lightweight convolutional network, computational consumption has not increased exponentially as imagined. Only with a similar computational amount can it achieve excellent performance. At the same time, ablation tests were performed on the feature extraction backbone (FEB) block based on the combination of GAN and transformer modules and the frequency domain and noise feature detection module (FFM) in the proposed model to further verify the efficiency of the proposed model. As shown in Table 3, in the performance comparison experiment based on the FF++ dataset with a relatively small number of videos, our model achieved an accuracy of 98.75%, while the area under the curve scored 99.43 and the F1 score was 98.67. Even when only the FEB module is used, the performance of our model is still slightly ahead of other models. In the test of the celeb-DF and DFDC datasets with relatively large data volumes, our model can still achieve better performance than other models.

**Figure 7:**
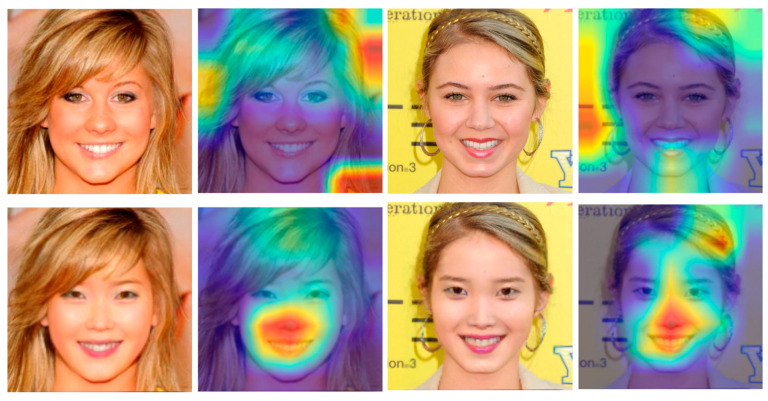
Feature detection comparison of real and fake images.

To further understand the details of the internal operation of the proposed model detection, the Grad-CAM [39] visualization tool is used here to visualize our model detection process. We used the data of the face-swapping model [40] from our previous study for the visualization of fake face detection, as shown in Figure 7, facing the original image, the model did not focus on specific features due to the absence of the fake operation feature region, but when facing the fake face image, the facial operation part was accurately detected by our model and the feature region was focused.

**Figure 8:**
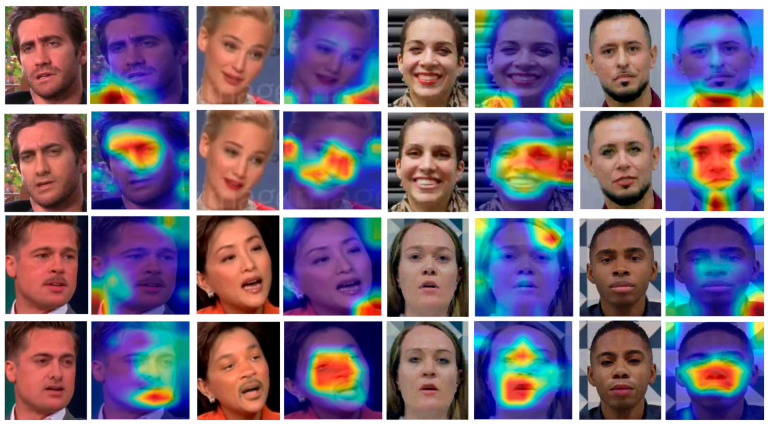
Comparison.

At the same time, we conducted a visual comparison of the detection of real and forged faces in the videos of the three datasets used, as shown in Figure 8. Facing various types of faces and facial features from different angles, our proposed model can accurately detect the forged operation trace area and make a quick judgment.

## 5. Discussion and Conclusions

In recent years, a large number of deepfake images have emerged, especially deepfake face images, which have brought privacy and security threats to residents and society. Although a large number of detection models have emerged at this stage, which has alleviated the detection pressure to a certain extent, there are still some neglected problems in these studies, such as insufficient model detection performance, inability to detect finely forged face images, poor generalization ability, and only being able to distinguish the authenticity of forged faces generated by specific datasets and specific models. To this end, in this paper, we designed a composite network with feature fusion to solve these problems of insufficient detection capabilities. The framework mainly consists of three modules, in which the reconstructed GAN and transformer modules are used to extract the main features of the image, the frequency domain and noise feature extraction modules extract hidden forgery traces, and all feature information is fused in the classification module. The final detection result is output through the SVM discriminator. After a large number of experimental verifications, our network is feasible, efficient, and more robust.

## Figures and Tables

**Table 1:** Information of fake datasets.

Database	Total Videos	Real Videos	Fake Videos
FaceForensics++ (FF++)	5000	1000	4000
Celeb-DF	6229	590	5639
DFDC	128,154	23,957	104,500

**Table 2:** Video dataset evaluation.

Model	ACC (%)	AUC (%)
TSN [35]	61.1	62.8
C3D [36]	64.3	65.4
I3D [37]	68.7	69.7
Two-stream [38]	70.10	72.35
Ours	78.91	84.06

**Table 3:** Cross-dataset evaluation.

Model	Dataset								
	FF++			Celeb-DF			DFDC		
	ACC	AUC	F1-Score	ACC	AUC	F1-Score	ACC	AUC	F1-Score
Xception	84.91	85.35	84.23	67.40	70.05	68.57	69.90	70.83	68.65
Wu et al.	85.45	85.82	83.23	68.94	72.90	70.39	73.02	77.11	74.10
Patch-DFD	86.83	88.20	87.01	72.85	75.38	73.15	75.06	78.93	75.33
TAN-GFD	90.37	92.51	90.35	83.71	87.17	84.59	84.63	86.79	84.05
fCNN	92.12	93.08	90.86	86.33	86.92	81.30	82.73	84.74	80.18
GANS	96.09	97.95	95.17	88.05	90.30	87.93	86.01	89.43	86.13
FEB (ours)	97.42	97.57	96.88	88.39	90.83	89.34	85.95	89.62	86.54
FEB+FFM (ours)	98.75	99.43	98.67	89.54	91.81	89.70	86.79	90.67	87.45

## Data Availability

All datasets utilized in this article are open-source and publicly available for researchers.
